# Investigation of local field enhancement near plain and shell-coated gold nanospheres for the optimization of surface enhanced spectroscopy†

**DOI:** 10.1039/d5ra03633j

**Published:** 2025-06-20

**Authors:** Ana-Maria Craciun, Daria Stoia, Aïcha Azziz, Simion Astilean, Monica Focsan, Marc Lamy de la Chapelle

**Affiliations:** a Nanobiophotonics and Laser Microspectroscopy Center, Interdisciplinary Research Institute in Bio-Nano-Sciences, Babes-Bolyai University 42 T. Laurian Str. Cluj-Napoca 400271 Romania; b Faculty of Physics, Babes-Bolyai University 1 M. Kogalniceanu Str. Cluj-Napoca 400084 Romania monica.iosin@ubbcluj.ro; c Institut des Molécules et Matériaux du Mans (IMMM – UMR6283), Université du Mans Avenue Olivier Messiaen Le Mans 72085 Cedex 9 France marc.lamydelachapelle@univ-lemans.fr

## Abstract

In this paper we perform a detailed and systematic investigation of electromagnetic field localization and enhancement, at different excitation wavelengths in the 520–640 nm domain, near spherical gold nanoparticles (AuNSs) of different sizes, using Finite-Difference Time-Domain (FDTD) simulations. We provide clear evidence of the size-dependent local electromagnetic field distribution and plasmon-dependent near-field intensity at the surface of plain and DNA-mimicking shell-coated individual AuNSs. This represents a crucial aspect which needs to be taken into consideration in the optimization of platforms based on AuNSs for plasmon-enhanced spectroscopies. Our set of FDTD simulations reveal useful insights regarding the extent of the spectral red-shift of the maximum electromagnetic field enhancement position relative to the localized surface plasmon resonance (LSPR) band, alongside an interesting AuNS size-dependent field enhancement variation in the 520–640 nm excitation range. Finally, we correlate some of the main important theoretical findings from FDTD simulations with experimental data from Surface Enhanced Raman Spectroscopy (SERS) and Metal Enhanced Fluorescence (MEF) assays based on particular types of plain and shell-coated AuNSs.

## Abstract 2

Systematic FDTD study of near-field distribution and intensity near plain and shell-coated AuNSs reveals crucial findings for surface enhanced spectroscopies.

## Graphical abstract

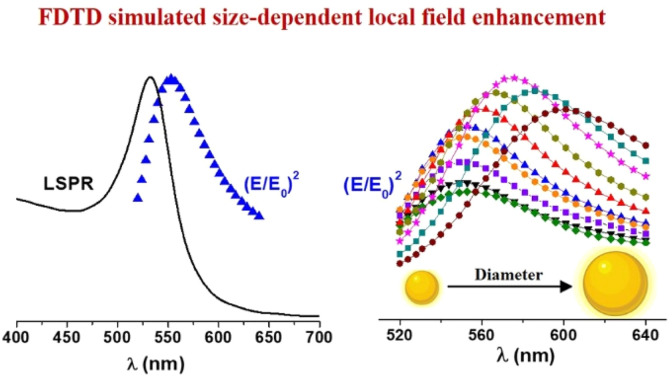

## 1. Introduction

The investigation of the electromagnetic field enhancement near plasmonic nanostructures represents a subject of intense current research essential for the advancement and optimization of technological plasmon-based applications, especially for improving the sensitivity and response in single molecular detection.

Gold nanoparticles (AuNPs) are extremely popular plasmonic NPs being considered as excellent candidates for surface-enhanced spectroscopies, such as Surface-Enhanced Raman Spectroscopy (SERS) or Metal-Enhanced Fluorescence (MEF). For instance, a primary important mechanism responsible for the enhancement of Raman signals in SERS is based on the intense electric field at the surface of plasmonic nanoparticles due to excitation of localized surface plasmon resonances (LSPR). In MEF on the other hand, the coupling of electronic excitations of fluorophores with surface plasmon resonances in the presence of an intense near field is able to boost the intensity of the fluorescence emission and increase the sensitivity of fluorescence-based detection of molecules positioned at a certain optimal distance from the metal surface. Therefore, it is crucially important to have a comprehensive understanding of the plasmon-dependent intensity and distribution of the local electromagnetic field around plain and molecular layer-coated AuNPs of various sizes and shapes.

Mathematical modelling methods are commonly used nowadays for predicting the optical properties of colloidal NPs and nanostructures. In order to study electromagnetic wave propagation in such materials, different numerical methods, both in time and frequency domains have been developed.^1–4^ Among various available numerical simulation methods, the Finite-Difference Time-Domain (FDTD) method is known to be the most efficient time-domain numerical 3D full-wave electromagnetic technique able to solve Maxwell's equations in complex geometries in order to provide reliable modelling of nanophotonic devices, processes and materials. FDTD method is extensively used to efficiently simulate light interacting with a variety of NPs, nanostructures and optical devices.^5^ As regards its use for plasmonic NPs, previous numerical simulations studies have revealed that the optical response and local field enhancement depends on the shape, size and material of NPs as well as other parameters such as the distance between them. FDTD simulations were successfully employed for investigating the properties of plasmonic NPs made of different materials. For instance, FDTD was used to study rhodium and platinum NPs for UV plasmonics,^6^ isolated, dimer and multimer aluminium nanostructures with plasmon resonances in deep UV^7^ or to investigate the plasmonic electric field enhancements and coupling effects in silver, Au and cooper NPs.^8^ However, FDTD is more often explored for simulating plasmonic effects and electromagnetic field distribution for AuNPs. For example, Cheng *et al.* used FDTD to investigate the influence of AuNP-size and NP–NP-spacing on the local electric field and extinction properties^9^ and Zhu *et al.* simulated the optical properties and electric field enhancement distribution for Au nanobipyramids modified with nanospheres.^10^ Similarly, Tira *et al.* analyzed the electromagnetic far- and near-field response of AuNPs organized in chain-like structures^11^ while others investigated the optical properties of single and coupled Au nanotriangles^12^ and different assembly of Au nanorods.^13^

In this paper, we have performed a comprehensive investigation of plasmon-dependent electromagnetic field distribution and enhancement around spherical AuNPs with diameters ranging from 15 to 100 nm, using numerical simulations based on the FDTD method. Spherical AuNPs represent one of the most intensely studied classes of plasmonic nanoparticles, employed for various applications, including assays based on surface enhanced spectroscopies. Therefore, our aim was to provide a comprehensive theoretical set of data on spherical AuNPs, over an extended range of sizes, serving as guidance for the optimization of enhanced spectroscopies, such as SERS and MEF, as function of particle size and excitation wavelength. The size-effects are carefully evaluated while the near-field and far-field intensities are simulated at multiple wavelengths in the 520–640 nm range, to assess the effect of plasmon resonant excitation in SERS applications.

Firstly, we investigate the relation between the spectral positions of maximum field enhancement and LSPR band. Although it is occasionally assumed that the maximum of the electromagnetic field at the surface of AuNP should correspond to the spectral position of the LSPR band or very close to it, experimental observations often show that the maximum SERS signal is obtained when using laser lines that are not resonant with the LSPR. This apparent discrepancy can be attributed to several factors, which we explore in detail in this work. Additionally, in view of MEF applications, we investigated the electromagnetic field distribution and intensity around the different-sized spherical AuNPs, encapsulated within dielectric shell of varying thickness to serve as spacer, at various excitation wavelengths. In particular, double-stranded DNA sequences can serve as high-precision nanometric spacers because the distance between two consecutive bases is known to be 0.34 nm. The covering shell with thickness varying between 1.7 and 6.7 nm, was particularly chosen to mimic DNA polyA–polyT decamer with 5, 10, 15 and 20 adenine bases, commonly used as spacers in MEF-based assays for imaging or detection purposes. We were then able to demonstrate the large influence of the molecular shell thickness and saturation on the field distribution and enhancement around the AuNSs.

Finally, we correlate some of the main theoretical findings on the size-dependent electromagnetic field near plain AuNSs with the results from demonstrative SERS experiments using 4-MBA (4-mercaptobenzoic acid) Raman reporter and 20, 50 and 100 nm diameter AuNSs performed at 638 nm excitation. Also, we compared the simulation results on local field enhancement near shell-coated AuNSs with MEF data for the particular case of 50 nm AuNSs coated with DNA polyA–polyT decamer with 5 and 20 adenine bases, labelled with Cyanine 5 fluorophore, at 630 nm excitation.

## 2. Materials and methods

### 2.1 FDTD simulations

All the simulations presented in this study were performed with Finite-Difference Time-Domain (FDTD) method using Ansys Lumerical FDTD software.^14^ We investigated the optical properties of individual plain spherical gold nanoparticles (AuNSs) with diameter ranging from 15 nm to 100 nm, placed in water (*n* = 1.33). We used the Johnson and Christy reference data for dielectric properties of gold.^15^ The mesh step size used varied between 0.2 nm and 0.4 nm as function of Au sphere size while as boundary conditions we used perfect matched layers (PMLs) approach on all directions. The absorption, scattering and extinction cross-sections of standalone AuNSs were simulated by using the total field scattered field (TFSF) method in which the particle is surrounded by two different monitor analysis groups. The absorption cross section is calculated from the analysis group located inside a vertically polarized TFSF source specifically designed for this type of method, where a non-periodic object is illuminated by a plane-wave. Similarly, the scattering cross section is calculated from the group located outside the TFSF source while the extinction cross section represents the sum of absorption and scattering results. The data for cross-sections were collected in the 400–1000 nm interval. The information on the electromagnetic field distribution and intensity were obtained from a field profile monitor placed through the center of the AuNS. We collected information on the field distribution around individual AuNS of varying diameter at 26 wavelengths in the 520–640 nm interval (4 nm step). We focus on this wavelength range because the plasmon resonances of spherical plasmonic AuNPs are within this range and our aim was to evaluate the shift between the plasmons and maximum enhancement. Near-field enhancement plots ((*E*/*E*_0_)^2^) display the intensity of the electric field (*E*) at a specific point near the AuNSs, relative to the intensity of the incident electric field (*E*_0_), expressed as a normalized value. In this case, we used a plane wave propagating perpendicular to the plane comprising the AuNS and incident electromagnetic field *E*_0_ polarized along the axis of the AuNS. As our goal was to evaluate the correlation between LSPR band position and maximum field enhancement, the main variable parameter was the diameter of the AuNS. The other simulation conditions were adjusted and optimized by preliminary tests. In the final part of our study, we also present the results regarding the extinction cross section and electromagnetic field distribution obtained for 15, 30 and 50 nm AuNS covered with a uniform dielectric layer with different thicknesses and a refractive index of 1.5, mimicking a spacer based on DNA polyA–polyT decamer (further denoted as simply DNA) with different number of adenine bases.

### 2.2 Experimental details

To estimate the SERS signal provided by AuNPs of different diameters, we use 4-mercaptobenzoicacid (4-MBA, Sigma-Aldrich, purity: 90%). This molecule is often used as a Raman probe in SERS experiments as it gives a large SERS signal. The 4-MBA included a thiol group that allows a grafting of the molecule at the gold surface through a S–Au bond. A 1 mM solution of 4-MBA was prepared by dissolving 3 mg of 4-MBA powder in 180 μl of 10% ethanol. The AuNSs with diameters of 20 nm, 50 nm, and 100 nm, supplied in 0.1 mM phosphate-buffered saline (PBS), were obtained from Thermo Scientific. According to the supplier, the AuNSs' concentrations were: 6.54 × 10^11^ part per ml (20 nm AuNSs), 3.51 × 10^10^ part per ml (50 nm AuNSs) and 3.84 × 10^9^ part per ml (100 nm AuNSs) which were converted to 1.09 × 10^−9^ M, 5.83 × 10^−10^ M and 6.38 × 10^−12^ M using Avogadro's number. The AuNSs were functionalized with the 4-MBA solution by incubating them overnight at room temperature (60 μl of MBA were added to 500 μl of AuNSs). The concentration of MBA (1 mM) was specifically chosen to get a number of MBA molecules largely higher than the number of AuNP (between 10^5^ to 2 × 10^7^ MBA molecules for one individual AuNP depending on the NP diameter) and consequently to ensure saturation of the AuNSs's surface and maximize the SERS signal. The following day, the suspension was centrifuged, and the supernatant containing unbound 4-MBA was removed and replaced with 0.01 mM PBS, which excludes any interference from the Raman signature of ethanol.

SERS measurements in solution were performed using a XploRA Raman spectrometer (Horiba Scientifics) equipped with a backscattered Raman signal detection system for liquid measurements (×4 lens, Horiba Scientifics). The measurements were carried out in a quartz cuvette containing 500 μl of solution. SERS spectra were recorded at 638 nm. The spectral resolution was of 2 cm^−1^. The SERS intensity was measured by integrating the 1075 cm^−1^ band area as this band is the most intense on the SERS spectrum. The obtained SERS intensities were normalized by the concentration of AuNSs in the solution.

Samples for MEF assays were based on AuNSs of 50 nm diameter synthesized using an optimized version of the protocol previously reported by Suarasan *et al.*^16^ ssDNA of adenine (polyA) and of thymine (polyT) were customized and acquired from Eurogentec (Seraing, Belgium). PolyA was specifically modified with thiol (–SH) modifier at 5′ to facilitate the successful functionalization at the surface of AuNSs and with the Cyanine 5 (Cy5) fluorophore at 3′. Both polyA and polyT were purified *via* the RP-HPLC method prior to being used. A 100 μM solution of 10^−4^ M polyA-Cy5 was prepared by dissolving the lyophilized stock in TE buffer, following the instructions provided in provided datasheet. Later, 50 nm AuNSs at a concentration of 6.79 × 10^11^ M, calculated using an extinction coefficient of 4.53 × 10^10^ L mol^−1^ cm^−1^, were functionalized with polyA-Cy5. For this, we incubated 150 μl of AuNSs solution with 10 μl of polyA-Cy5 (5 or 20 bases, 100 μM) at room temperature for 2 hours, followed by overnight incubation at 4 °C. The used concentration ensures a maximum loading and saturation of the employed AuNSs' surface with polyA-Cy5 strands. Two individual samples prepared, one functionalized with polyA of 5 bases (5B polyA), and the other with 20 bases (20B polyA).

The fluorescence signal from these samples was used as reference in MEF experiments. Further, a hybridization protocol using complementary ssDNA samples, specifically 5B and 20B polyT at the same initial concentration of 10^−4^ M, induced the formation of the DNA polyA–polyT decamers at the surface of AuNSs. The hybridization protocol consisted in a 1 h room temperature incubation, followed by an overnight incubation at 4 °C. Considering that each A–T base pair fully extended has around 0.34 nm, the lengths of the double-stranded polyA–polyT DNA are approximated 1.7 nm (5B) and 6.7 nm (20B), which brings the Cy5 molecules at different distances from the metal surface.

The fluorescence emission measurements presented in this work were performed with a Jasco FP6500 spectrofluorometer from Jasco International Co., Ltd (Tokyo, Japan). The spectra were acquired at room temperature with a resolution of 1 nm, employing a Xenon lamp (150 W) as the excitation source. Emission spectra were recorded at 630 nm excitation (1 × 3 nm excitation and emission bandwidths), specific for the Cy5 fluorophore. Quartz glass cuvettes of 5 × 5 mm from Hellma were used. All fluorescence emission spectra were recorded and analysed with the Spectra Manager and OriginPro 9.0.

## 3. Results and discussion

### 3.1. Study of field enhancement around individual AuNSs

**Fig. 1:**
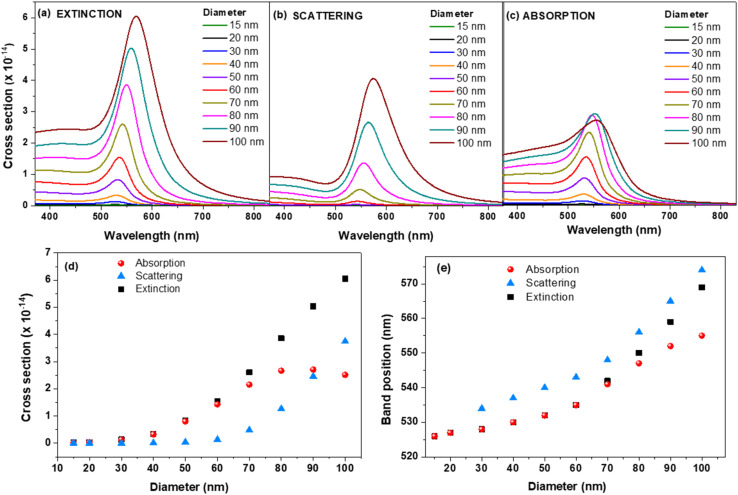
FDTD simulated extinction (a), scattering (b) and absorption (c) cross sections of plain AuNS of different diameters. Variation of simulated cross sections (d) of extinction, scattering and absorption and their band positions (e) as function of AuNS′ diameter.

To investigate the plasmonic activity and contribution of AuNPs to the electromagnetic enhancement phenomenon in surface enhanced spectroscopies, our first goal was to evaluate the connection between the LSPR band position and the field enhancement at different wavelengths in the 520–640 nm interval in the case of single AuNSs of varying diameter, the most common and explored type of AuNPs. Thus, we first simulated the optical response of individual AuNS of different sizes in aqueous media. Fig. 1 presents the extinction, scattering and absorption cross-sections of single AuNS with diameter in the 15–100 nm range as function of wavelength, as well as the variation of cross sections and LSPR band positions as function of diameter.

Extinction cross sections measure the strength of electromagnetic wave interaction with the plasmonic nanoparticles and account for the total amount of light energy that is removed from the incident beam due to scattering and absorption by the NP. It is a well-known that the intensity and spectral position of LSPR band depends on the factors affecting the electron charge density on the surface of the NP surface such as the metal type, particle size, shape or dielectric constant of the surrounding medium.^17^ As expected, in our case the position of FDTD computed LSPR band moves to higher wavelength with the increase of diameter from 526 nm for 15 nm AuNS to 569 nm for 100 nm AuNS (see Table 1 and Fig. 1).

**Table 1:** Statistics on the different values obtained from simulation for plain individual AuNS of different diameters between 15 and 100 nm^a^

*d* (nm)	*λ*_LSPR_ (nm)	*λ*_S_ (nm)	*λ*_A_ (nm)	*S* (%)	*A* (%)	*λ*_max_(*E*/*E*_0_)^2^ (nm)	Δ*λ* (nm)	(*E*/*E*_0_)^2^ intensity at		
								520 nm	Max	640 nm
15	526	—	526	<1	>99	553	27	17.1	29.6	12.1
20	527	—	527	<1	>99	550	23	21.2	32.0	16.4
30	528	534	528	1.6	98.4	551	23	23.1	37.5	18.1
40	530	537	530	5.0	95.0	552	22	24.6	44.4	19.9
50	532	540	532	6.7	93.3	553	21	24.6	47.3	21.0
60	535	543	535	13.7	86.3	560	25	21.2	51.8	24.2
70	542	548	541	19.2	80.8	566	25	18.7	56.3	28.1
80	550	556	547	25.4	74.6	575	28	16.2	60.3	33.7
90	559	565	552	39.6	60.4	586	34	12.7	56.8	37.8
100	569	574	555	42.2	57.8	600	45	10.1	51.8	42.1

a *d* = diameter of AuNS; *S* = scattering; *A* = absorption; *λ*_A_ = spectral position of absorption band; *λ*_S_ = spectral position of scattering band; Δ*λ* = *λ*_max_(*E*/*E*_0_)^2^ − *λ*__LSPR__; *S* (%) and *A* (%) were calculated using the integrated intensities.

The same trend is followed by the absorption and scattering bands as displayed in the graphic from Fig. 1e. We can see that the up to 60 nm, the extinction and absorption wavelengths overlap. After 60 nm, the extinction band gets more and more red-shifted compared to the absorption one, which seems to be heading to a plateau and is also observed in the plot of the cross section as function of diameter (Fig. 1d). On the other hand, the scattering band continuously red-shifts with the size. The contribution of scattering and absorption with the increase of size are displayed in Fig. S1† and summarized in Table 1.

The contributions of absorption and scattering are strongly correlated with the size of AuNS. For example, the scattering for 15 nm and 20 nm AuNS is almost insignificant (<1%), which means that most of the incoming light gets absorbed. On the other hand, as the diameter increase beyond 30 nm, the contribution of scattering starts to be more and more important, reaching for instance a 42.2% contribution for 100 nm diameter. Our findings rely on the well-known skin depth effect which governs the interaction of metallic NPs with incoming electromagnetic light and heat transfer.^18^ Typically, metallic skin depths are few tens of nm at optical wavelengths, effectively limiting the interaction volume between the light and NP to a small shell near the surface of the particle. The depth of the skin layer, *δ* = *λ*/(2π*ε*′′), where *ε*′′ is the imaginary part of the refractive index of the material, assuming unit relative magnetic permeability, is directly proportional with the wavelength of the light but inversely proportional with the square root of the materials' permittivity as shown in Fig. S2a.† Based on the physical skin depth concept, a particle of diameter *d* < *δ* exhibits a trapping force proportional to the total particle volume, while particles with *d* > *δ* exhibit a trapping force proportional to the shell volume.^18,19^

According to this effect, for NPs with *d* < *δ*, the electromagnetic field penetrates the entire particle. In this case, the entire NP contributes to the plasmonic resonance, mainly due to absorption and very little to scattering (see the values of scattering in Table 1). On the other hand, for NPs much larger than the skin depth, that is approximately *d* > 60–70 nm, according to the values obtained for the skin depth at 530–550 nm (Fig. S2a†), the electromagnetic field does not penetrate in the whole volume of the AuNP and is confined mainly at a surface region of the NP. In this case, only the electrons located in the shell determined by skin depth contribute significantly to the plasmonic resonance. At these sizes there is a balance between the scattering, which controls the field enhancement and the absorption, which dictates the heating in the shell. This might also explain the observed plateau after 70 nm diameter (see Fig. 1d), where the absorption efficiency is maximized,^20^ as well as the fact that for AuNPs with *d* > 80 nm, the scattering dominates absorption in the LSPR spectra. The variation of the extinction cross section with diameter follows the results from Mie theory obtained by Jain *et al.* for 20, 40 and 80 nm AuNSs.^21^ We compared the FDTD data on extinction with those predicted by Mie theory.^22^ As shown in Fig. S3,† the extinction cross sections obtained with FDTD are in good agreement with the data from Mie theory.

**Fig. 2:**
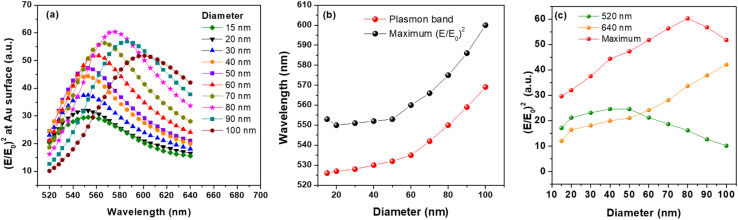
(a) Value of (*E*/*E*_0_)^2^ near plain AuNS of different diameters at different wavelengths in the 520–640 nm interval, (b) position of plasmon band and maximum (*E*/*E*_0_)^2^ as function of AuNS' diameter, (c) value of (*E*/*E*_0_)^2^ near AuNS at 520 nm, 640 nm and at the position of maximum enhancement, as function of diameter.

Thereafter, we simulated the near electromagnetic field around individual AuNS at different wavelengths in the 520–640 nm interval and plotted the resulting maximum (*E*/*E*_0_)^2^ values, from the surface of the corresponding AuNS, on the same graphic (Fig. 2a). We observe that the (*E*/*E*_0_)^2^*vs.* wavelength plot for each size exhibits a maximum value at a position which red-shifts with the increase of size. As shown in Fig. 2b, the trend observed for the position of maximum (*E*/*E*_0_)^2^ (black dots) resembles that of plasmon band position (red dots). Up to the size of 50 nm, the plasmon bands red-shift quite slightly with the increase of size. After this, the red-shifts becomes more and more pronounced with the increase of size from 50 nm to 100 nm. One can also clearly notice that the position of the maximum field enhancement is systematically red-shifted compared to the position of the LSPR regardless the diameter. The shift between both positions is nearly constant between 20 and 30 nm for all diameters.

The existence of such red-shifts between the plasmon resonance and the maximum near-field enhancement as well as its dependence on the size of the noble-metal particles has been frequently pointed out in the literature.^23–25^ However, the values reported for the shifts between these peaks are considerably lower than the values that we obtained with FDTD simulations. For instance, the shifts we have calculated for AuNSs with diameters between 15–100 nm using the simple analytical harmonic oscillator model previously proposed to explain the observed phenomenon,^24^ are only in the 1–5 nm range.

Interestingly, when plotting the field enhancement value as function of AuNS's size (Fig. 2c – red dots) we observe that the 80 nm AuNS exhibits the most intense (*E*/*E*_0_)^2^ from all analysed samples. This could be a consequence of a particular combination between absorption process, as dictated by the skin depth, and energy loss through damping which is related to the permittivity of Au at the wavelength corresponding to maximum (*E*/*E*_0_)^2^ and start to increase at wavelength higher than 600 nm.

The occurrence of a maximal electric field intensity around AuNSs at a nearby redder wavelength than the LSPR was also reported recently by other researchers.^26^ Similarly, based on a set of previous results obtained based on an electrodynamic model including finite-size effects,^27^ the authors assign the observed size dependence of the near electric field intensity to a combination of radiative damping, surface light scattering and dynamic depolarization effect.

The position at which the maximum enhancement (*E*/*E*_0_)^2^ is obtained for each size together with its value are also summarized in Table 1. We also extracted the values of field intensity at 520 nm and 640 nm and displayed them in the same plot with the maximum values for each size (Fig. 2c). For a better visibility, values are summarized in Table 1. For example, a maximum of 60.3 intensity enhancement is obtained at 575 nm for 80 nm AuNS while at 520 nm and 640 nm the maximum field enhancements are 24.6 and 42.1 for 50 nm AuNS and 100 nm AuNS, respectively. Thus, when using 520 nm excitation wavelength for SERS experiments using AuNSs it is most suited to adjust their size around 40–50 nm. On the other hand, our simulations show that 100 nm AuNSs or even larger are more suited in experiments using 640 nm excitation wavelength. It should be noted that these two wavelengths are off-resonance relative to the LSPR position of any nanoparticle size.

The first wavelength is located at higher energy and remains away from the maximum as the nanoparticle size increases and primarily induces absorption, while the second wavelength, located at lower energy, results in a more balanced contribution of absorption and scattering and thus a better potential for SERS applications. A similar trend was observed by Starowicz *et al.* at 514 and 633 nm in their FDTD study on plasmonic properties of metal NPs of different sizes and shapes^28^ and Cheng and co-workers at 510 nm for 10–60 nm AuNs.^9^ Our results are also in good agreement with the experimental findings of S. Hong and X. Li.^29^ According to them, for the same number of NPs, the enhancement factor generated from the AuNSs under excitation at 647 nm, increases as the size of NP increases.

**Fig. 3:**
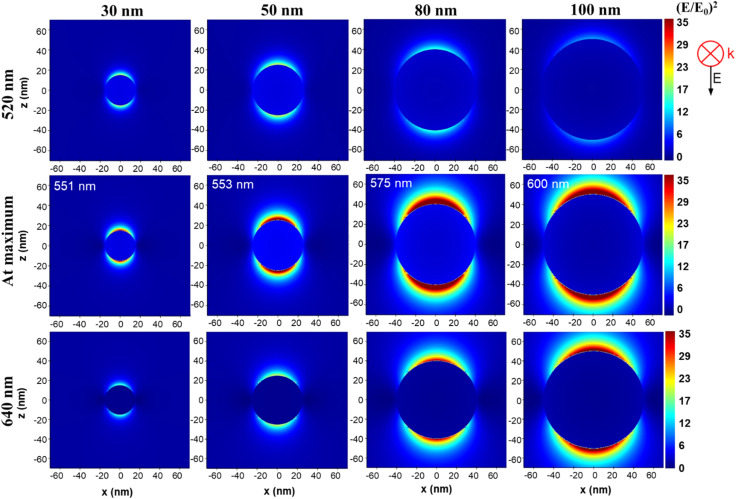
(*E*/*E*_0_)^2^ distribution near the surface of a AuNS with diameter of 30, 50, 80 and 100 nm at 520 nm, 640 nm and at the position of maximum field enhancement, at the wavelengths indicated in each image, according to values from Tabel 1.

For exemplification, we have presented in Fig. 3 the distribution of (*E*/*E*_0_)^2^ at 520 nm, 640 and at the position of maximum enhancement value for 4 selected sizes in the 15–100 nm range. We can observe that we have two areas of electromagnetic field enhancement for each individual AuNS, at their equators and this appearance is due to using of polarized light in simulation. However, the electromagnetic field intensity varies significantly with the size and wavelength of observation as previously mentioned. Specifically, we observe that we have the most intense field at 520 nm for the 50 nm AuNS while at 640 nm the intensity of the field constantly increases with the size of AuNS. On the other hand, we observe that in the middle row, where we displayed the distribution of field at maximum enhancement position, the most intense field is generated at the surface of 80 nm AuNS, closely followed by 100 nm AuNS.

Next, we also investigated near-field decay as we move away from the surface of the AuNS. This aspect is important to evaluate as the different (bio)molecules, proteins or other biological molecules investigated through SERS have different dimensions and therefore the extent of signal enhancement is directly correlated with the intensity of the field at a certain position from the surface of the NP. Fig. S4† shows the (*E*/*E*_0_)^2^ decay near plain AuNS of different diameter at the position of maximum enhancement as well as at the two selected marginal wavelengths, 520 and 640 nm. We observe in all cases that the (*E*/*E*_0_)^2^ decay slope gets less and less steep as the size of the AuNS increases. Due to this effect, we observe for 520 nm, for instance, where the superposition of the decay curves is more obvious, that the relationship between the (*E*/*E*_0_)^2^ value and size of the AuNS is changing with the distance from Au surface. This effect is also visible for the decay of (*E*/*E*_0_)^2^ at maximum field enhancement (Fig. S4b†) for 80–100 nm sizes when the field intensity starts to decrease and the decays start to superpose.

**Fig. 4:**
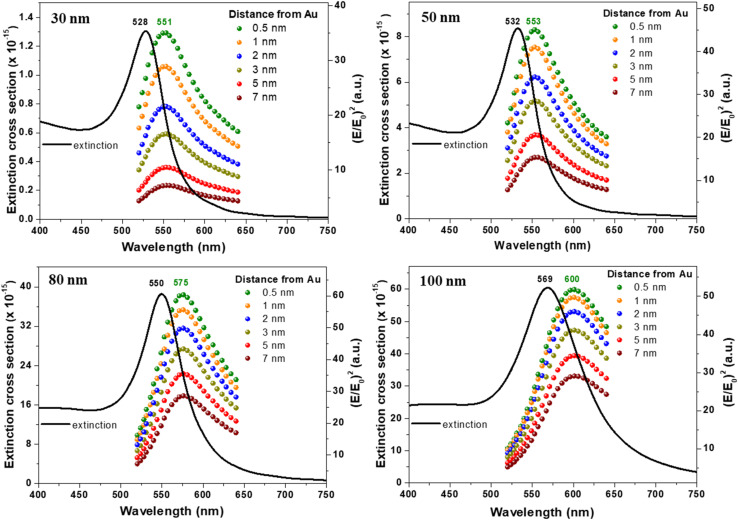
Superposition of extinction band and (*E*/*E*_0_)^2^ at different distances from Au surface in the 520–640 nm interval for AuNS of 30, 50, 80 and 100 nm diameter.

Moreover, we were interested to evaluate the position at which the maximum field enhancement occurs for each of the investigates sizes. For this we have plotted in onto the same graphic the extinction band and the representation of (*E*/*E*_0_)^2^*vs.* wavelength at different distances from the Au surface for 4 selected sizes (Fig. 4). Similar plots for the other sizes are presented in Fig. S5† while the wavelengths at which the plasmon resonance and maximum enhancement occur are also summarized in Table 1. Interestingly, we found out that the maximum field enhancement occurs at a position which is red-shifted compared to the position of the LSPR band. The red-shifts are over 20 nm for all sizes while the shape of (*E*/*E*_0_)^2^*vs.* wavelength and the position of the maximum enhancement does not modify with the distance form Au surface.

The observed red-shift between the resonance wavelength and the position of maximum near field enhancement can be explained by the energy loss through damping which occurs in plasmonic materials. The damping is notably related with the absorption and one can notice that the field enhancement is largely decreased at lower wavelength range of the plasmon resonance where the absorption is the highest. The absorption will then reduce the field enhancement efficiency inducing a red-shift of the maximum of the field enhancement. One can also notice that when the absorption band become wider for diameter larger than 80 nm, the field enhancement is also affected as it decreases compared to the one observed for a diameter of 80 nm. The damping mechanisms, such as electron–phonon scattering and interactions with the surrounding medium, dissipate energy and broaden the LSPR band. The damping causes a phase shift between the driving force of the incident light and the restoring force due to the polarization of the nanoparticle, shifting the maximum electric field to a slightly longer wavelength. The ability of a nanoparticle to enhance the optical near field is directly related to the imaginary part of the dielectric function. The imaginary part describes the losses encountered in polarizing the material. Thus, figures of merit based on the ratio between the real part and the imaginary part of the dielectric function of a material, have been used to compare the plasmonic capabilities of different materials, independently of the size of nanoparticle.^30^ Similarly, Lalisse and co-workers introduced a more refined dimensionless parameter based on this ratio to quantify the efficiency of different plasmonic materials for near-field enhancement.^31^ Due to the previously mentioned material-related energy loss, a 21 nm spectral shift is obtained between the resonance wavelength and the wavelength of the radiated near-field, which correlates with our results. Also, the observed red-shift is in direct relation with the well-known frequency shift between the near-field and far-field plasmonic resonances.^24,32^ Nevertheless, compared to other materials, Au and silver are the two most often used for plasmonic applications due to their relatively low loss in the visible and NIR ranges. Therefore, our results prove that for optimized enhancement in SERS assays it is important to use an excitation wavelength which not only does not match the LSPR band of the employed AuNSs, but it is considerably red-shifted with respect to the plasmonic band.

**Fig. 5:**
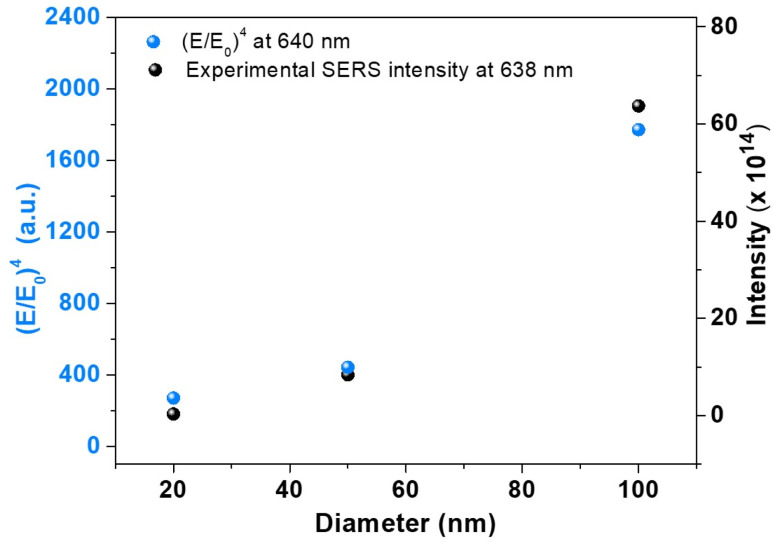
Comparison between the experimental integrated SERS intensity of the 1075 cm^−1^ band of the 4-MBA obtained for AuNSs of different sizes, under 638 nm, and the FDTD simulated (*E*/*E*_0_)^4^ at 640 nm.

To have an experimental validation of our simulation findings, we recorded the SERS spectra of 4-MBA Raman reporter using 20, 50 and 100 nm AuNSs. Fig. 5 presents the superposition of the experimental SERS intensities obtained under 638 nm excitation, with the FDTD simulated (*E*/*E*_0_)^4^ values at 640 nm for the same diameters. Typically, the good correlation between the magnitude of SERS intensity and the maximum of field enhancement reveals the so-called *E*^4^ dependence in SERS which accounts for both excitation and emission, each of them by a power of 2. It has been shown that, despite the fact that it is almost always an approximation, due to its simplicity, it is in many cases a useful estimation of the order of the enhancement factor.^33^ The SERS intensities were normalised to the concentrations of AuNSs in the solution. We observe that the SERS signal increases with the diameter, similar to previous demonstration where the number of AuNPs was kept constant.^29^ More importantly, we observe that both the SERS signal and (*E*/*E*_0_)^4^ increase with the diameter of the AuNs which proves the strong correlation between the two parameters and confirms the size-dependent field enhancement.

The small differences between the two parameters could arise from the inhomogeneity of the experimentally employed AuNSs compared to the ones used in simulations together size-dependent plasmon-assisted enhancement of excitation and scattering processes in experimental assays.

### 3.2 Study of field enhancement around shell-coated individual AuNSs

It is known that the LSPR of noble-metal NPs can enhance the fluorescence signals under certain conditions, such as proper spectral overlapping between SPR and fluorescence bands and a separating distance larger than ∼8–10 nm between the NP and the fluorophore, resulting in metal-enhanced fluorescence (MEF).^34,35^ Depending on the separation distance, a strong competition between the two opposite effects, nonradiative fluorescence quenching and plasmonic fluorescence enhancement, occurs. A lot of effort is devoted nowadays to find optimal anti-quenching spacers able to promote plasmon-assisted enhanced fluorescence phenomenon. Therefore, in MEF applications, it is extremely important to evaluate and understand the electromagnetic field intensity decay from the metal surface in the presence of a physical spacer with variable thickness. Generally, spacers are made of non-metallic materials such as polymers^36,37^ or dielectric materials,^16,38^ but DNA molecules^39,40^ are also considered suitable candidates as spacer materials for fluorescence enhancement. Such nano-systems based on AuNPs coupled with DNA-labeled with fluorophores^41^ or other emissive species such as nanoclusters,^42^ carbon dots^43^ or quantum dots^44^ continue to be developed and optimized for various fluorescence-based applications such as imaging and sensing, including MEF assays.

Having this in mind, we further evaluated theoretically the electromagnetic field distribution and decay at the surface of an individual AuNS covered with a DNA shell – imitating compact spacer as function of AuNS's diameter and spacer thickness. We chose for exemplification, from the 15–100 nm range of diameter to perform the FDTD simulations for 15, 30 and 50 nm AuNSs. As for the spacer shell, we chose the thicknesses of 1.7, 3.3, 5 and 6.7 nm corresponding to the lengths of the DNA strand with 5, 10, 15 and 20 adenine bases (polyA), respectively, hybridised with its complementary strand (5, 10, 15 or 20 thymine, polyT), known as polyA–polyT decamer (further denoted as simply DNA) frequently used in various experimental studies as spacer for the controlled separation of fluorescent species from the plasmonic surfaces of NPs or nanostructures.

First, we evaluated the modifications induced by the compact shell covering the AuNS, on the extinction, scattering and absorption cross sections, as function of AuNS's diameter and shell thickness (Table 2).

**Table 2:** Statistics on the different values obtained from simulation for 15, 30 and 50 nm shell-coated individual AuNS^a^

*d* (nm)	Shell thickness (nm)	*λ*_LSPR_ (nm)	*λ*_S_ (nm)	*λ*_A_ (nm)	*λ*_max_(*E*/*E*_0_)^2^ (nm)	Δ*λ* (nm)	(*E*/*E*_0_)^2^ intensity at		
							520 nm	Max	640 nm
15	1.7	532	—	532	549	17	8.3	15.6	7.2
	3.3	534	—	534	565	31	4.5	8.4	5.1
	5	536	—	536	569	33	2.5	4.9	3.2
	6.7	537	—	537	567	30	2.1	3.9	2.8
30	1.7	531	536	531	550	19	14.0	25.1	11.2
	3.3	533	538	533	552	19	8.5	17.0	7.8
	5	535	540	535	554	19	6.2	13.1	5.9
	6.7	536	543	536	555	19	5.8	11.4	5.2
50	1.7	535	542	535	557	22	15.0	34.2	15.7
	3.3	537	544	537	559	22	10.3	25.9	12.1
	5	538	545	538	561	23	8.0	21.9	10.5
	6.7	540	547	540	563	23	6.4	18.8	9.2

a *d* = diameter of AuNS; *S* = scattering; *A* = absorption; *λ*_A_ = spectral position of absorption band; *λ*_S_ = spectral position of scattering band; Δ*λ* = *λ*_max_(*E*/*E*_0_)^2^ − *λ*_LSPR_.

**Fig. 6:**
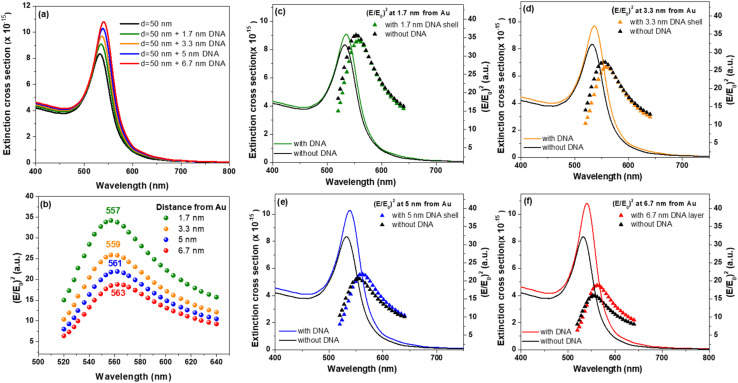
(a) FDTD simulated extinction cross-sections of 50 nm diameter AuNS coated with DNA of different thickness. (b) Value of (*E*/*E*_0_)^2^ at the DNA shell–water interface at different wavelengths in the 520–640 nm interval. (c)–(f) Superposition of extinction band corresponding to 50 nm AuNS without/with DNA shell and (*E*/*E*_0_)^2^ at 1.7, 3.3, 5 and 6.7 nm from Au surface in the absence and presence of DNA shell, in the 520–640 nm interval.

The LSPR and absorption bands red-shifts in the presence of DNA shell, while this shift increases proportionally with the thickness of the shell due to refractive index modification. Also, the presence of a coating shell increases the light scattering and absorption leading to an increase of extinction proportional with shell thickness (Fig. 6a and S6–S8†).^45^ By plotting the values obtained for (*E*/*E*_0_)^2^ at the DNA shell–water interface at different wavelengths in the 520–640 nm interval, for the different DNA shell thicknesses, we observed a red-shift of the maximum (*E*/*E*_0_)^2^ wavelength position from 557 nm to 562 nm together with the overall decrease of (*E*/*E*_0_)^2^ intensity with the increase of shell thickness (Fig. 6b).

The observed decrease is similar for 15 and 30 nm AuNSs (Fig. S9†). The (*E*/*E*_0_)^2^ intensity is almost 5 times lower at 6.7 nm from the surface of a 15 nm AuNS compared to a 50 nm AuNS, which suggests that bigger AuNSs can promote a higher electromagnetic field, and thus better chances of enhancing the fluorescence signal of emissive species placed at such distance from the metallic surface. Additionally, as the plots in Fig. 6c–f suggest, it is also crucial to have an excitation wavelength which is non-resonant with the LSPR band, but is red-shifted compared to it. The position at which the maximum (*E*/*E*_0_)^2^ intensity is obtained in the presence of DNA shell is located at larger wavelengths compared to the position obtained for plain AuNSs.

As shown in the plots from Fig. 6c–f these red-shifts are similar with those observed for the LSPR band in the presence on DNA shell (black curves compared to coloured curves). Thus, the presence of a DNA shell as well as the variation of its thickness impacts the electromagnetic field enhancement at the shell border which further impacts the degree of signal enhancement corresponding to any possible DNA-attached molecules. The (*E*/*E*_0_)^2^*vs.* wavelength plots also reveal an interesting aspect regarding the intensity of (*E*/*E*_0_)^2^ at a certain distance from the Au surface in the presence and absence of DNA shell. The presence of a DNA shell of 1.7 nm or 3.3 nm thickness induces a small decrease of (*E*/*E*_0_)^2^, whereas in the case of 5 nm and 6.7 nm shell we observe an increase of the field enhancement at the shell–water interface.

**Fig. 7:**
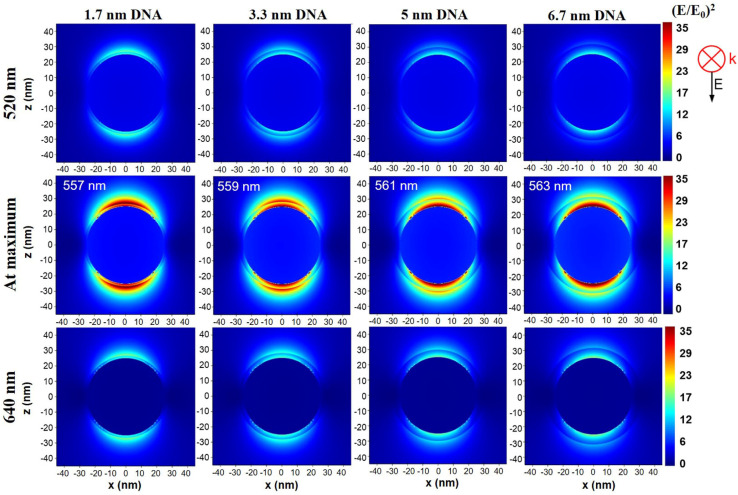
(*E*/*E*_0_)^2^ distribution near the surface of a 50 nm diameter AuNS coated with DNA of different thickness at 520 nm, 640 nm and at the position of maximum field enhancement, at the wavelengths indicated in each image, according to values from Tabel 2.

This electromagnetic field modifications occurring at the shell–water border are also visible in Fig. 7 and S10–S13,† in which the distribution of (*E*/*E*_0_)^2^ near AuNSs in the presence of the DNA shell at 520 nm, 640 nm and at the position of maximum value, are displayed. In all the presented images, we observe a decrease of the electromagnetic field from Au surface towards the shell–water border where a “jump” of the field intensity occurs due to refractive index modification.

This sudden increase is also visible in the (*E*/*E*_0_)^2^ decay plots presented in Fig. S14.† With black dotted lines are superposed the decays obtained at the three excitation wavelengths, for 50 nm AuNS in the absence of a DNA shell. The enhancement of the electromagnetic field directly above the DNA shell is in good agreement with the observations and model of Neeves and Birnboim.^46^

This sharp increase of the field intensity induced by the refractive index change at the dielectric layer/surrounding medium interface was previously observed for polyelectrolyte-coated AuNSs^16^ and polyvinylpyrrolidone-coated AuNSs.^47^ Often, AuNPs are coated with dielectric shells for biocompatibilization and functionalization purposes yet suffer from the weakening of local electric field enhancement. Like our findings, Deng *et al.* also demonstrated that, instead of being weakened, the local electric fields can be enhanced by using an appropriate high refractive index dielectric coating.^48^ According to their findings, the high refractive index shell owns strong Mie resonances that can participate in the bonding plasmon hybridization being able to avoid diminishing of light absorption, while sustaining a great leap of the local electric fields reaching the surface. The two mechanisms can work synergistically to offset the decay of the local electric fields inside the shell. Similar effects are effective in providing Shell-Isolated Nanoparticle-Enhanced Raman Spectroscopy (SHINERS) in SERS spectroscopy.^49^ In our case, as the DNA shell confines the electromagnetic field around the nanoparticle, the dielectric boundary conditions for the electric field must be satisfied at the DNA–water interface. This requires that the two normal components of the electric displacement field vectors D should be equal at the DNA shell–water interface, which means that *ε*_DNA_ × *E*_DNA_ = *ε*_water_ × *E*_water_ or alternatively *n*_DNA_^2^ × *E*_DNA_ = *n*_water_^2^ × *E*_water_ where *n*_DNA_ and *n*_water_ are the bulk refractive indexes of DNA and water, respectively. However, using the FDTD-calculated *E*-field values and the bulk refractive index of the DNA shell (*n*_DNA_ = 1.5) and water (*n* = 1.33), this relationship is not satisfied (see Table S1†). In fact, the above dielectric boundary condition between two dielectric layers (DNA *vs.* water) is valid for infinitely large media (semi-infinite spaces from both sides of the interface), which, obviously, is not our case. In fact, near the interface, we have not only a nanometric DNA shell confining the electromagnetic field but also a plasmonic nanoparticle under excitation, necessitating the treatment of the system as an ensemble of two interacting materials. Therefore, we employ the concept of effective refractive index, which originates from the effective medium theory currently employed in designing new metamaterial^50^ and has been previously introduced by Astilean and co-workers^51^ to interpret the light transmission of subwavelength metallic gratings with very small apertures. This theory allows us to replace an inhomogeneous optical material, such a thin dielectric shell coating a metallic nanoparticle with an “homogeneous layer” made of hypothetical material that exhibits equivalent optical properties. In particular the “effective refractive index” required to satisfy the electromagnetic boundary conditions, can be calculated as 
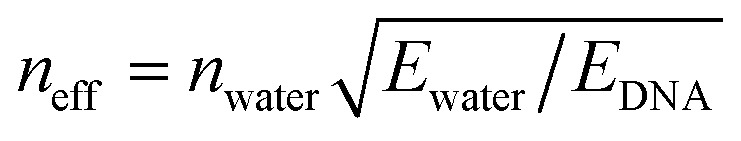
. More interestingly, we have in Fig. S14† a clear proof that this “effective refractive index” is strongly dependent on the shell thickness, thus on the distance from the metallic surface, which is an interesting and valuable effect to report. As shown in Table S1,† the value of *n*_eff_ changes with the thickness of the shell. It increases from a shell of 1.7 up to 5 nm and then decreases for a shell of 6.7 nm. Particularly, at all three analysed wavelengths, the leap of the (*E*/*E*_0_)^2^ at the DNA shell–water interface increases with the decrease of the DNA shell thickness, as consequence of “effective refractive index” modulation by the presence of the nanoparticle. This effect can then be tuned by varying the thickness of the dielectric layer, leading to a significant increase of the enhancement of the localized surface plasmon resonance at the surface of the dielectric layer. Over the distance of about 20 nm the electromagnetic influence of nanoparticle onto interface can be considered negligible and therefore the interface DNA–water can be treated electromagnetically as two large dielectric layers with bulk refractive indexes. We believe this concept helps us accurately predict or optimize the electromagnetic response of many nanometer-scale plasmonic–dielectric light couplers. Using effective refractive index as a tool to predict the amplification of electromagnetic field at layered plasmonic–dielectric interface offers a significant advantage for the analysis and development of new SERS and LSPR biosensors, providing a deeper physical insight on the effect of surface coverage by molecules deposited on the surface of nanoparticles.

We also evaluated the impact of a partially saturated molecular layer on field enhancement. Thus, we performed additional simulations for AuNS with *d* = 50 nm covered by a 6.7 nm dielectric layer with different refractive indices between 1.372 and 1.53, corresponding to various degrees of coverage, from ∼25% degree of surface coverage (*n* = 1.372) up to a dense DNA layer (*n* = 1.53). The (*E*/*E*_0_)^2^ decay plots are presented in Fig. S15.† We observed that the jump increases with the increase of layer refractive index, thus with the degree of molecular layer saturation. Specifically, at 520 nm the saturation of the molecular layer indices a more pronounced variation of the electromagnetic field close to the Au surface, within the layer, at 640 nm the field the variations are similar in the two areas, while at the wavelength of the maximum enhancement, we observe that the field varies significantly out of the molecular layer, effect which can be exploited in MEF assays by the appropriate choice of molecular layer and/or saturation level. In particular, at maximum field enhancement for example, the higher the refractive index of the spacer, the higher is the field enhancement for an analyte located at around 6.7 nm from the nanoparticle surface. Similar observations regarding the possibility of enhancing the local electric fields using appropriate high refractive index dielectric coatings have been reported.^48,52^

Finally, to evaluate the field enhancement at the shell/air interface with the variations of the refractive index of the surrounding medium, we performed additional simulations for AuNS with *d* = 50 nm covered with a 6.7 nm layer (*n* = 1.5) placed in media of different refractive index in the 1.3–1.39 range. As shown in Fig. S16,† the refractive index of the medium has a more pronounced effect on the field within the molecular layer, while at 520 nm the effect occurs outside the shell, however the effect is less pronounced. Therefore, the electromagnetic field felt by a molecule situated at approximately 6.7 nm from the Au surface would be the same, regardless the refractive index of the surrounding media.

**Fig. 8:**
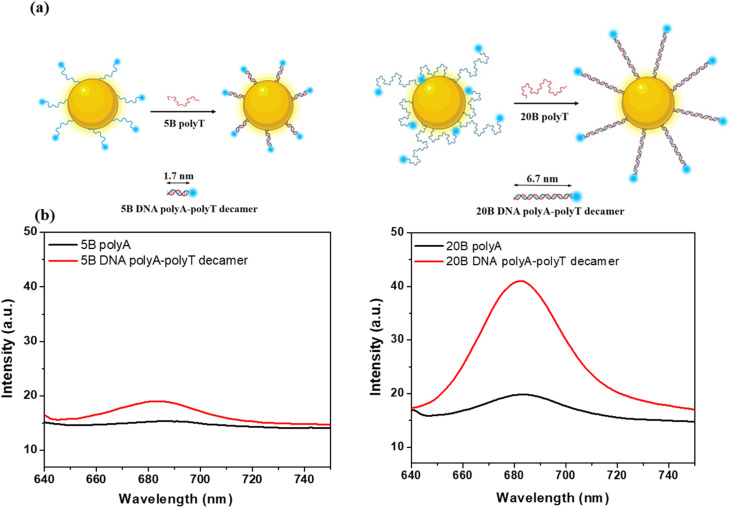
(a) Schematic representation of the envisaged conformational changes occurring after the hybridization of Cy5 – labelled polyA with polyT, at the surface of AuNSs. (b) Experimental enhancement of Cy5 molecule fluorescence emission by 50 nm AuNS using 5B and 20B polyA alone or 5B and 20B DNA polyA–polyT decamers. Excitation at 630 nm.

To assess the contribution of the local field intensity to MEF phenomenon, we measured experimentally the enhancement of Cyanine 5 (Cy5) molecule emission when coupled with 50 nm AuNSs. To change the distance between the Cy5 and the AuNSs' surface, the Cy5 was inserted at the extremity of a DNA strand, that acted as a spacer. We used two different DNA sequences: HS-AAAAA-Cy5 (5B polyA) and HS-AAAAAAAAAAAAAAAAAAAA-Cy5 (20B polyA). Each strand includes a thiol group to be grafted at the surface of the AuNPSs. After grafting the polyA, we hybridized them with their complementary strand (5B polyT or 20B polyT) to form polyA–polyT decamers. In this latter case, the lengths of 5B and 20B polyA–polyT decamers are respectively 1.7 nm and 6.7 nm. As references we used the emission spectra of Cy5-labelled 5B and 20B polyA strands attached to 50 nm AuNSs. The experimental fluorescence spectra obtained under excitation at 630 nm are presented in Fig. 8b. We observe in both cases an increase of Cy5 fluorescence emission after hybridization with polyT and formation of DNA polyA–polyT decamers. For 5B, as the separation distance is only around 1.7 nm, the quenching is still strong, thus the emission enhancement is very low, despite the large local field enhancement obtained from simulation (Fig. S10†). On the other hand, in the case of 20B strand, we assume that the hybridization induces a conformation change of Cy5-labelled polyA strands which are forced to elongate and adopt mostly a standing configuration moving thus the Cy5 molecules away from the Au surface, at about 6.7 nm (Fig. 8a).

Such positioning is far enough to reduce the quenching but still in an area of electromagnetic field enhancement (see Fig. S13†), enough to promote MEF with a factor of almost 1.7. Even though the FDTD simulations prove that the local field enhancement at 640 nm decreases exponentially from the Au surface, we obtained experimentally a larger MEF factor at 6.7 nm from the surface, when using 20B DNA polyA–polyT decamer, due to favourable positioning of Cy5 molecules. This clearly proves that not only the local field intensity, directly connected with the thickness and refractive index of the shell acting as spacer, controls the MEF effect but also the optimal coupling distance between plasmon and molecular dipoles. Based on simulation results, even higher signal enhancement is expected to be obtained by using higher refractive index coatings due to an enhancement of the local electric fields, which indicates a clear correlation between the strength of near-field and dielectric shell properties. Therefore, we believe that our results can be of significant importance for the development and optimization of plasmonic platforms based on DNA-labelled AuNSs for applications relying on surface enhanced spectroscopies, such as SERS and MEF.

## 4. Conclusions

To conclude, we performed FDTD simulations to investigate the electromagnetic field localization and enhancement near plain and shell-covered individual AuNSs. Our data reveal new insights regarding the influence of size and shell thickness, valuable for the optimization of surface enhanced spectroscopies. In particular, we report a significant spectral red-shift of the maximum electromagnetic field enhancement position relative to the LSPR band, alongside an interesting size-dependent field enhancement variation in the 520–640 nm excitation range. Additionally, we reveal a sharp shell thickness/saturation – dependent increase of the field intensity occurring at the dielectric layer/surrounding medium interface. We comprehensively explain the observed phenomenon using a model based on effective refractive index change at the interface. We believe that the elaborated set of discussions regarding multiple phenomena such as skin-depth, field variation at interface due to refractive index, field variation with shell thickness and refractive index of shell/medium at different distances from metal enhance the novelty of our manuscript and prove the relevance of our finding in the optimisation of surface enhanced spectroscopies. Finally, in order to verify our main theoretical findings and envisage the importance of local field enhancement and variation as function of various factors, we performed demonstrative SERS and MEF assays based on plain and DNA polyA–polyT decamers-coated AuNSs, respectively.

## Conflicts of interest

There are no conflicts to declare.

## Supplementary Material

RA-015-D5RA03633J-s001

## Data Availability

The data supporting this article have been included as part of the ESI.†
