# Supplementary material for: Investigation of local field enhancement near plain and shell-coated gold nanospheres for the optimization of surface enhanced spectroscopy
Source: RSC Adv. 2025 Jun 20;15(26):20848–62. doi: 10.1039/d5ra03633j (PMC12179859; doi:10.1039/d5ra03633j)
Supplement: RA-015-D5RA03633J-s001 [file RA-015-D5RA03633J-s001.pdf]

Supplementary Information (SI) for RSC Advances.
This journal is © The Royal Society of Chemistry 2025

Electronic Supplementary Information

Investigation of local field enhancement near plain and shell-coated gold nanospheres for the optimization of surface enhanced spectroscopy

Ana-Maria Craciun[1], Daria Stoia[1,2], Aïcha Azziz[3], Simion Astilean[1,2], Monica Focsan[1,2,*], Marc Lamy de la Chapelle[1,3,*]

[1] Nanobiophotonics and Laser Microspectroscopy Center, Interdisciplinary Research Institute in Bio-Nano-Sciences, Babes-Bolyai University, 42 T. Laurian Str., 400271, Cluj-Napoca, Romania

[2] Faculty of Physics, Babes-Bolyai University, 1 M. Kogalniceanu str., 400084, Cluj-Napoca, Romania

[3] Institut des Molécules et Matériaux du Mans (IMMM - UMR6283), Université du Mans, avenue Olivier Messiaen, 72085 Cedex 9 Le Mans, France

**Corresponding authors*: marc.lamydelachapelle@univ-lemans.fr; monica.iosin@ubbcluj.ro

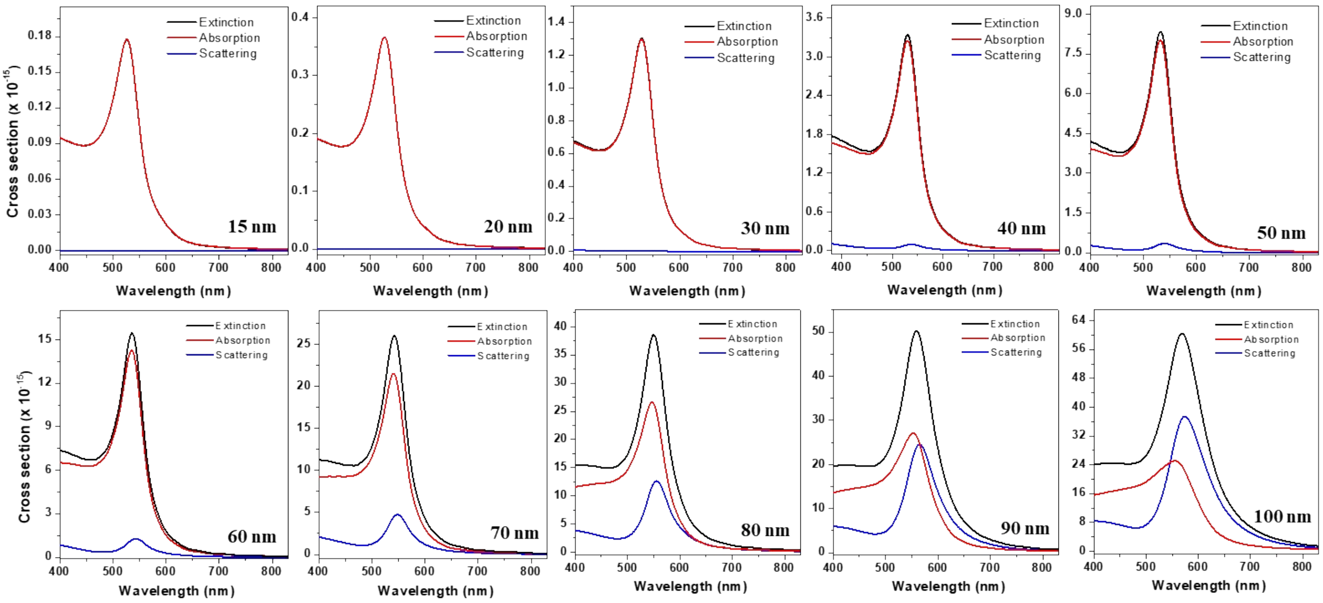

Figure S1. Superposition of extinction, absorption and scattering spectra corresponding to an individual plain AuNS with diameter in the 15 – 100 nm interval.

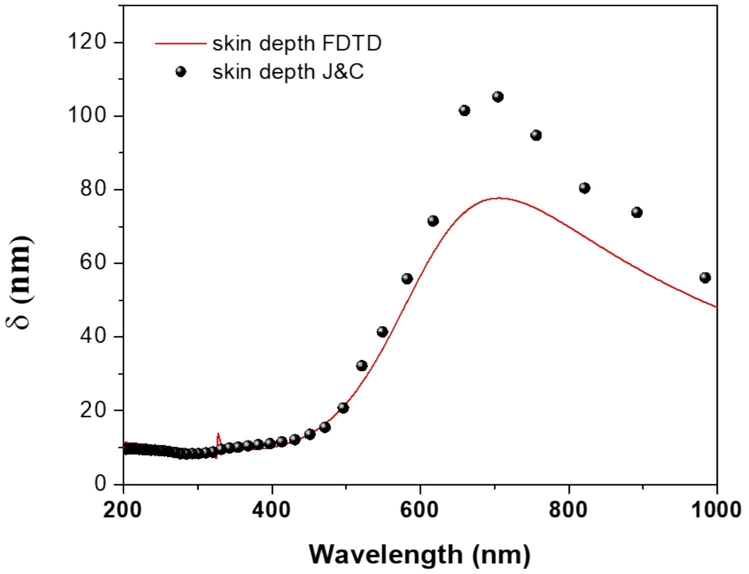

	530 nm	540 nm	550 nm	560 nm	570 nm
δ_{FDTD} (nm)	30.5	33.5	37.3	41.0	45.2

Figure S2. Skin depth for Au calculated with the formula $\delta = \lambda/(2\pi\varepsilon'')$, where ε'' is the imaginary part of the refractive index of the material, for ε'' taken from FDTD model used and Johnson & Christy (J&C) data. Table with the values extracted from data based on FDTD model at 530, 540, 550, 560 and 570 nm.

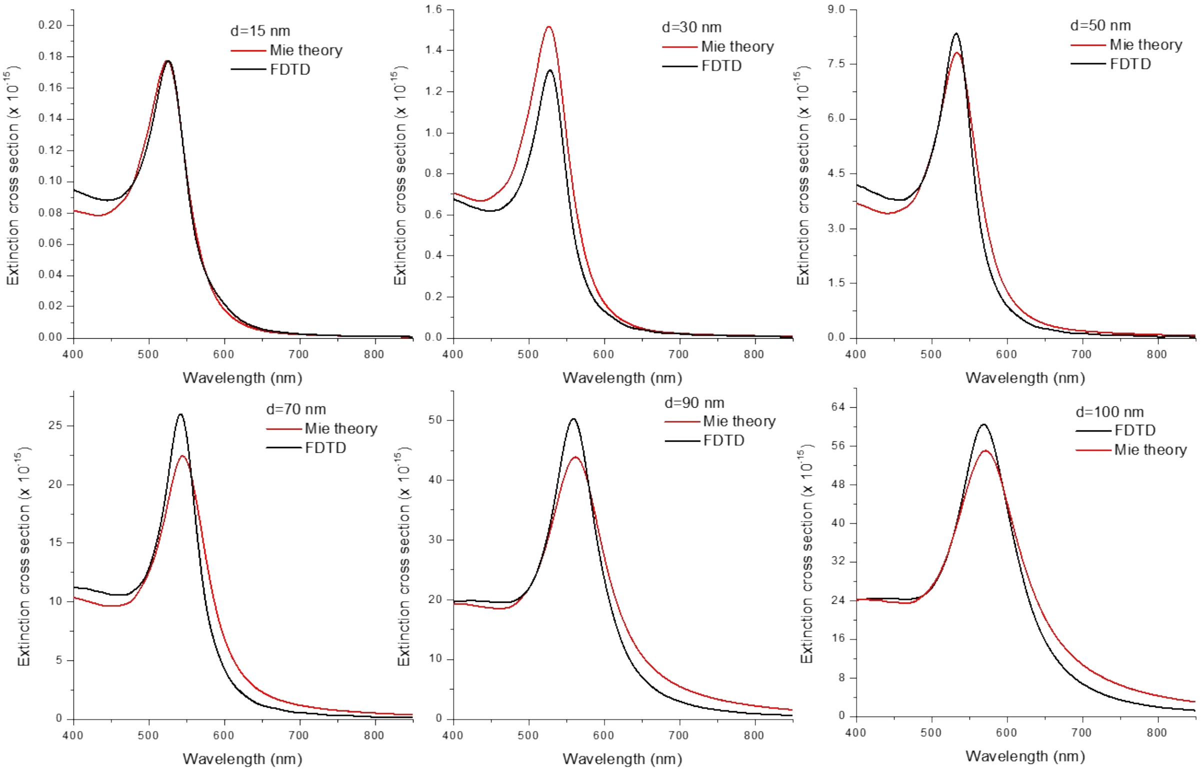

Figure S3. Comparison between the extinction cross-sections obtained with FDTD and Mie theory for AuNS of different diameters.

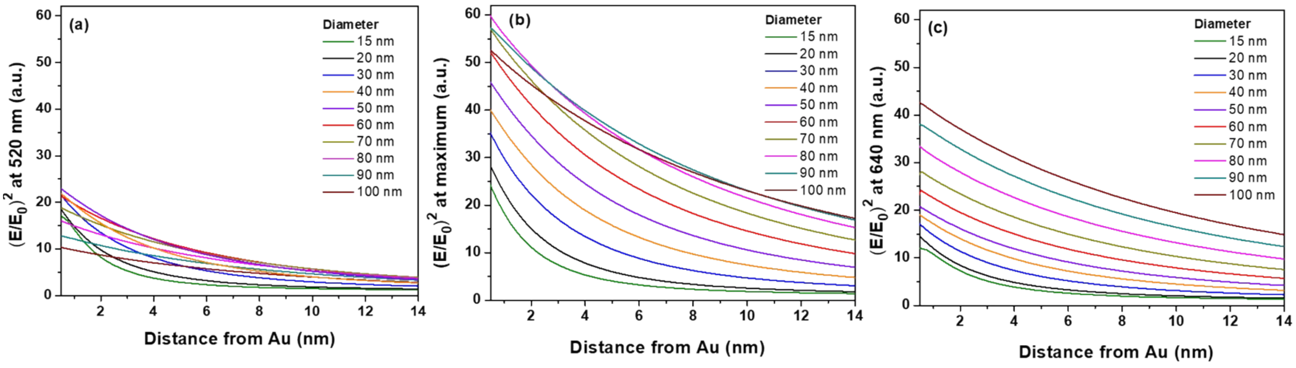

Figure S4. $(E/E_0)^2$ decay near plain AuNS of different diameter at 520 nm (a), at 640 nm (c) and at the position of maximum field enhancement, according to values from Tabel 1 (b).

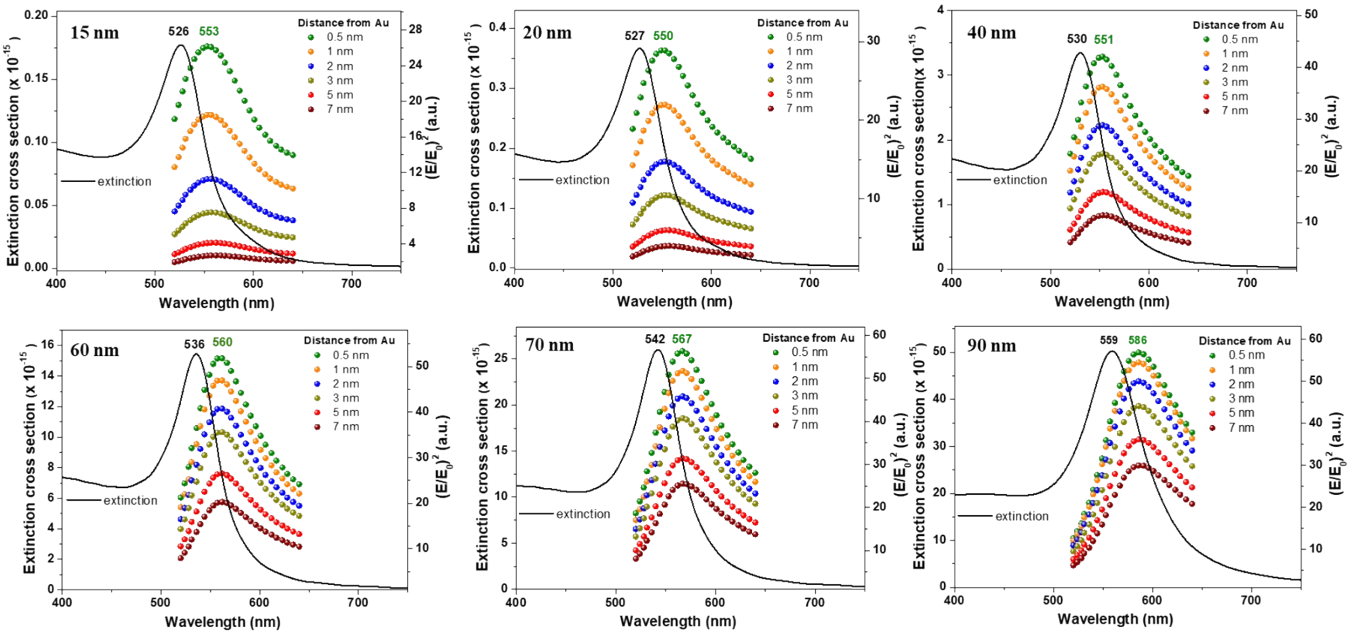

Figure S5. Superposition of extinction band and $(E/E_0)^2$ at different distances from Au surface in the 520 – 640 nm interval for AuNS of 15, 20, 40, 60, 70 and 90 nm diameter.

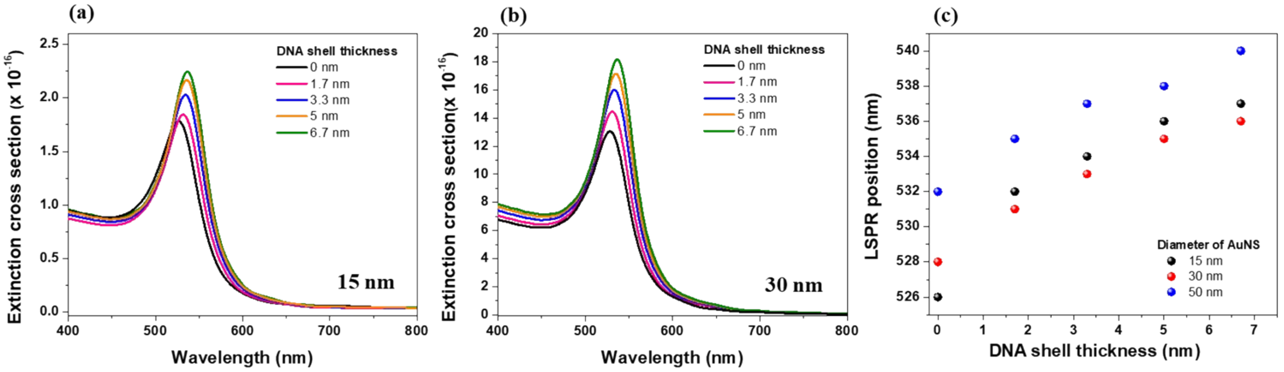

Figure S6. FDTD simulated extinction cross-sections of 15 nm (a) and 30 nm (b) diameter AuNS coated with DNA of different thickness. (c) LSPR band position as function of DNA shell thickness, for individual AuNSs with 15, 30 and 50 nm diameter.

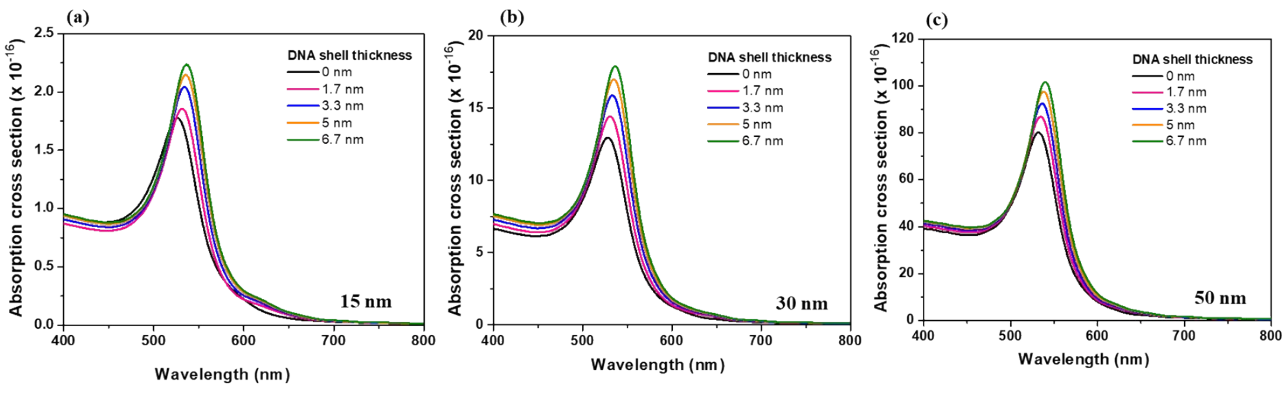

Figure S7. FDTD simulated absorption cross-sections of 15, 30 and 50 nm AuNS coated with DNA of different thickness.

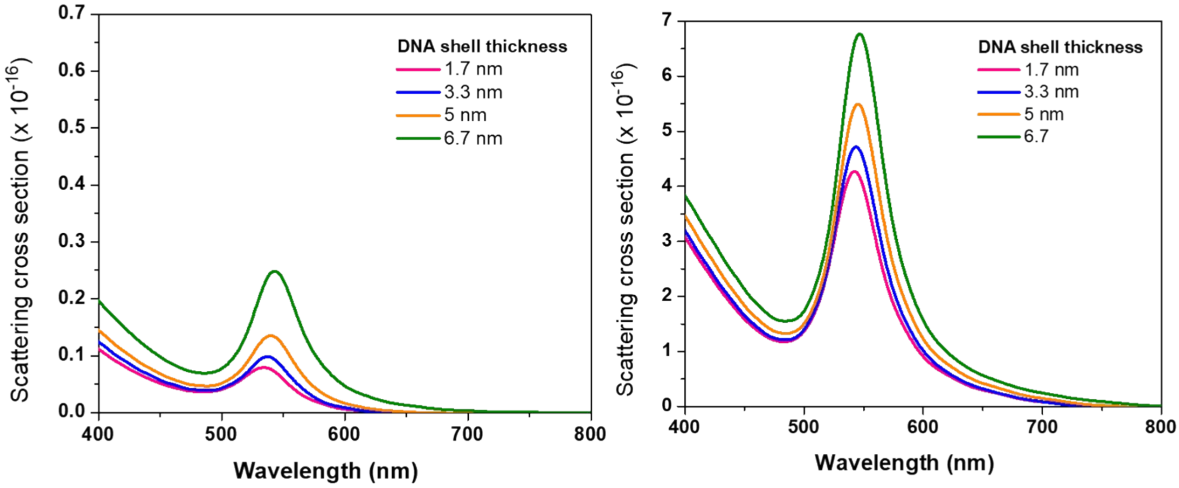

Figure S8. FDTD simulated scattering cross-sections of 30 and 50 nm AuNS coated with DNA of different thickness.

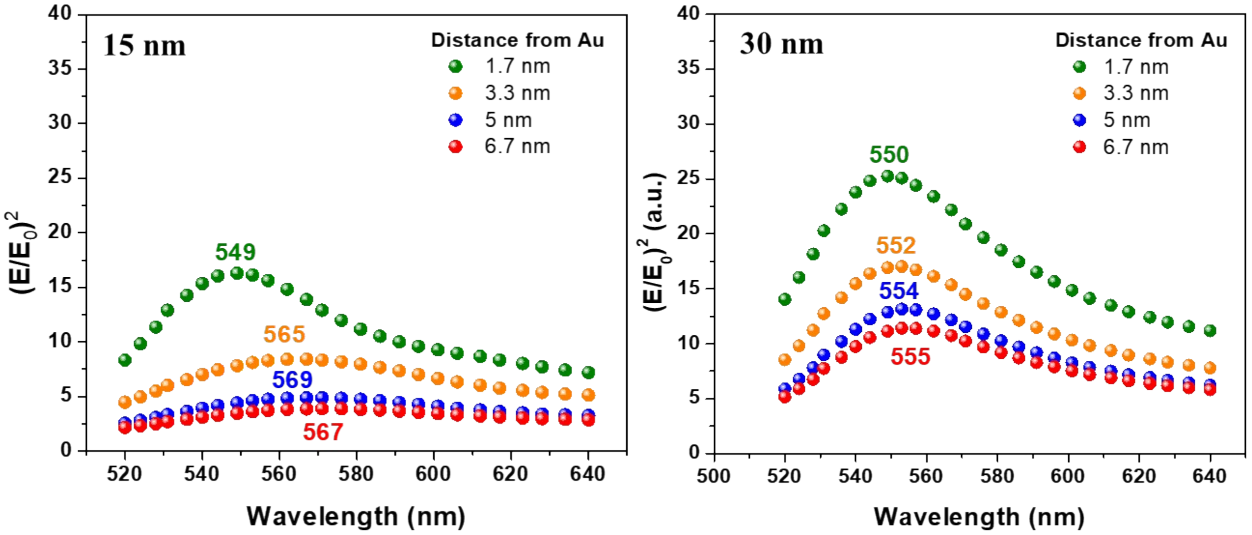

Figure S9. $(E/E_0)^2$ at the DNA layer - air interface at different wavelengths in the 520 – 640 nm interval for AuNS with 15 and 30 nm diameter.

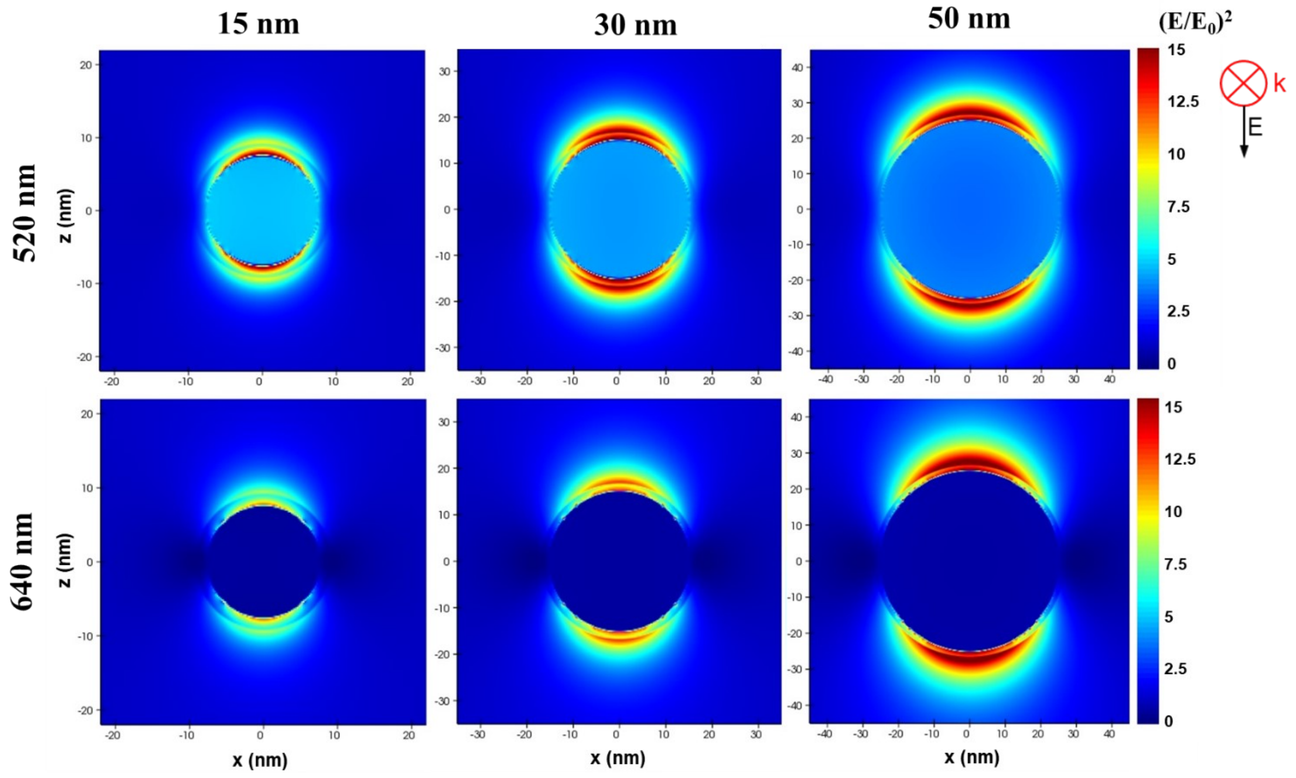

Figure S10. $(E/E_0)^2$ distribution around 15, 30 and 50 nm nm diameter AuNS coated with 1.7 nm DNA shell, at 520 nm and 640 nm.

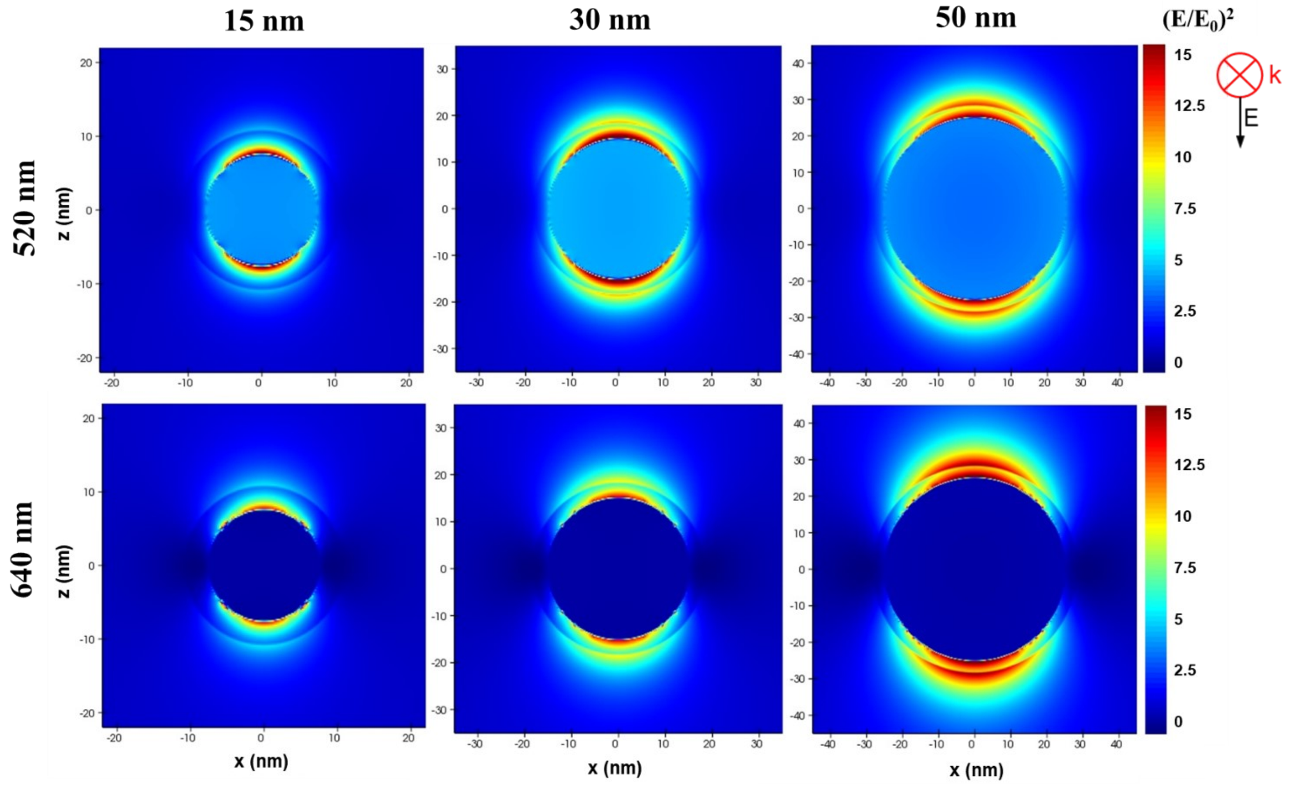

Figure S11. $(E/E_0)^2$ distribution around 15, 30 and 50 nm nm diameter AuNS coated with 3.3 nm DNA shell, at 520 nm and 640 nm.

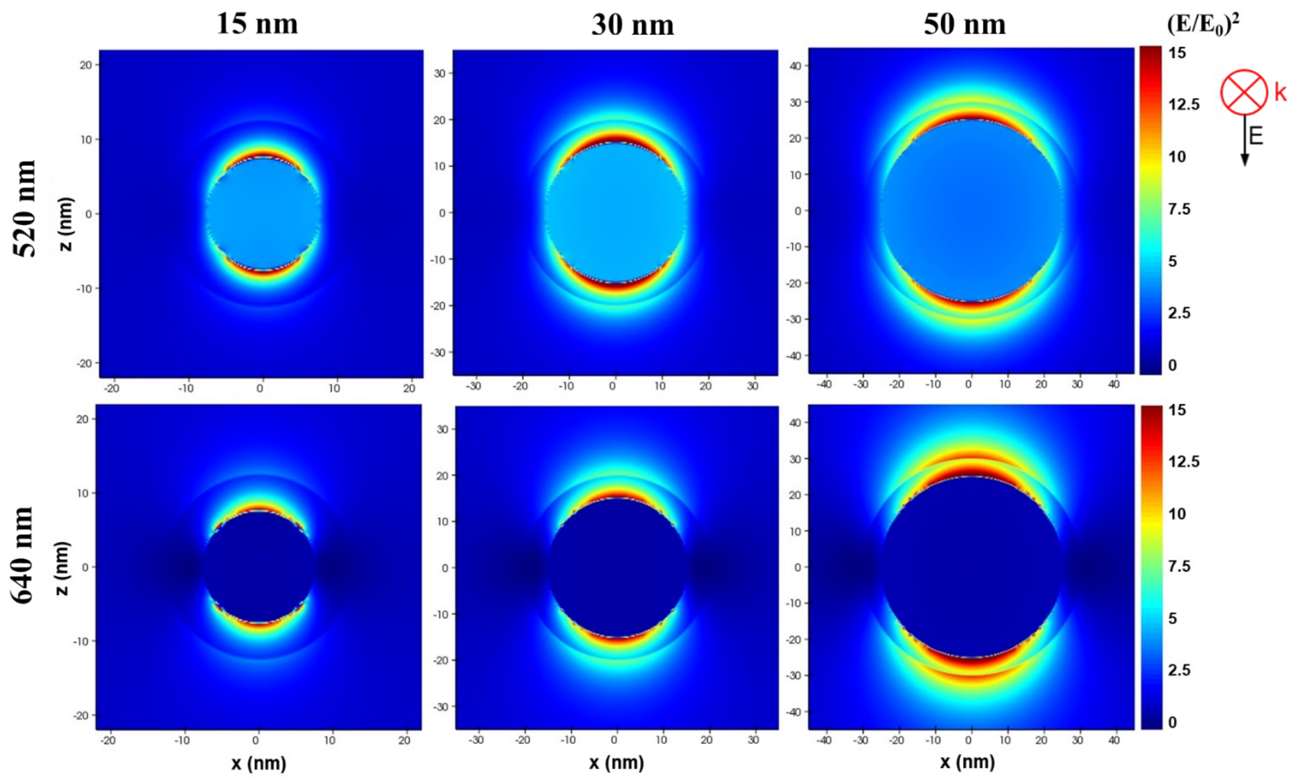

Figure S12. $(E/E_0)^2$ distribution around 15, 30 and 50 nm nm diameter AuNS coated with 5 nm DNA shell, at 520 nm and 640 nm.

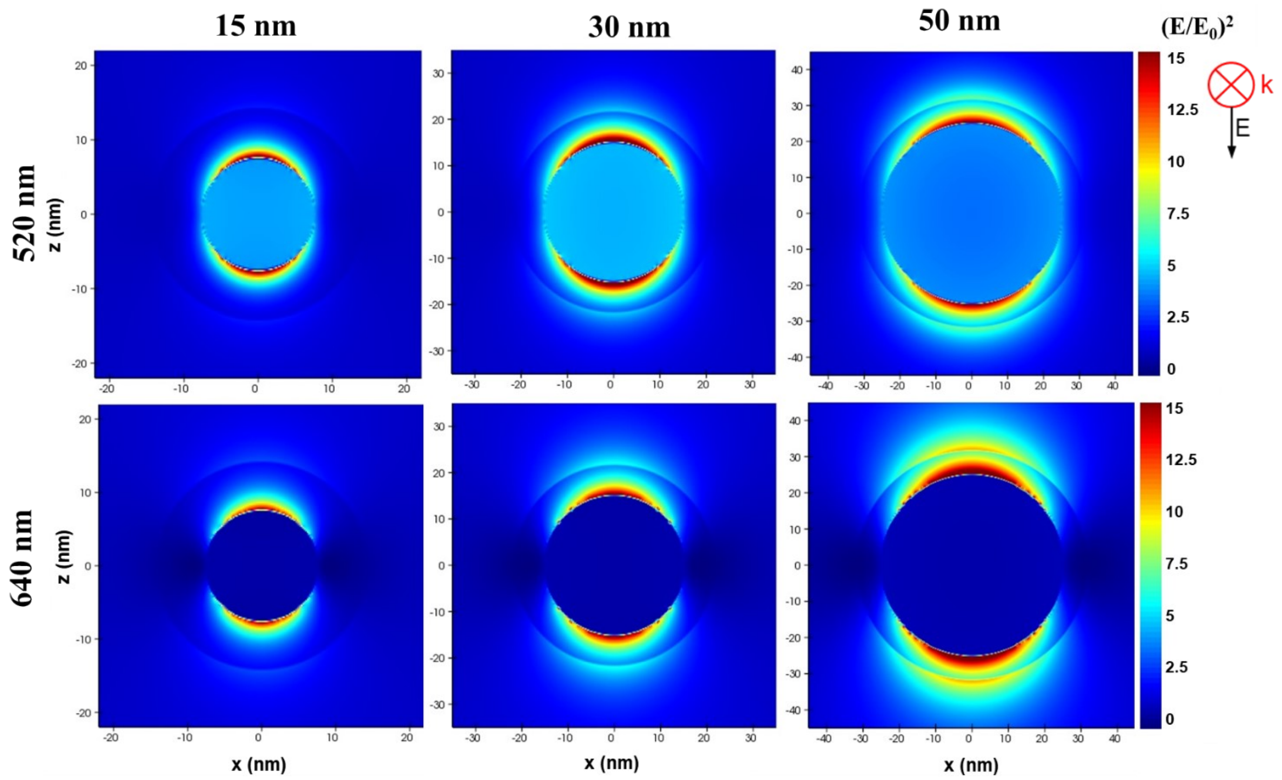

Figure S13. $(E/E_0)^2$ distribution around 15, 30 and 50 nm nm diameter AuNS coated with 6.7 nm DNA shell, at 520 nm and 640 nm.

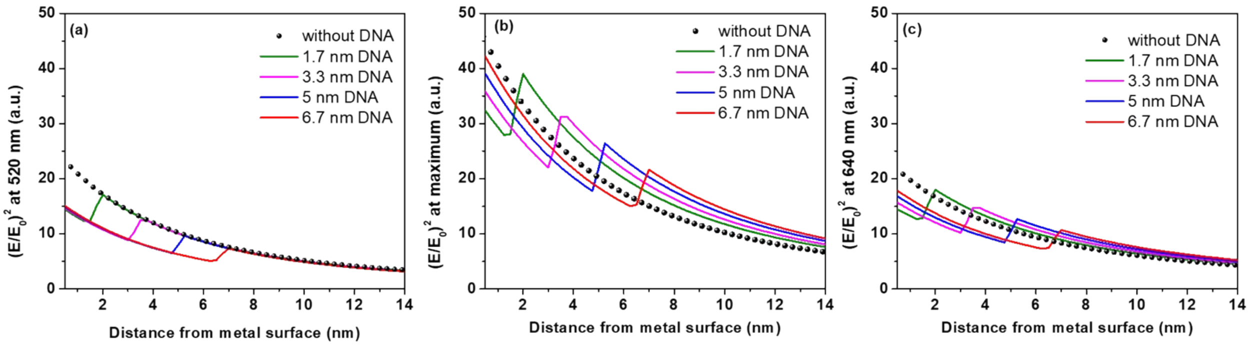

Figure S14. Decay of $(E/E_0)^2$ near plain and DNA-coated 50 nm diameter AuNS at 520 nm (a), 640 nm (c) and at the position of maximum field enhancement for each case: at 553 nm for plain AuNS and at 557 nm, 559 nm, 561 nm and 562 nm for the AuNS + DNA shell of 1.7 nm, 3.3 nm, 5 nm and 6.7 nm, respectively (b).

Table S1. E/E_0 values obtained at 520 nm, 640 nm and at maximum at the water-DNA interfaces, for 50 nm AuNS, along with the values calculated for $n^2 \times E$ ($n_{water} = 1.33$, $n_{DNA} = 1.5$)

	**E/E_0 at 520 nm**		**E/E_0 at max**		**E/E_0 at 640 nm**	
	In DNA	In water	In DNA	In water	In DNA	In water
1.7 nm	3.49	4.12	5.29	6.24	3.56	4.23
3.3 nm	2.98	3.56	4.71	5.60	3.18	3.83
5 nm	2.57	3.08	4.22	5.13	2.88	3.56
6.7 nm	2.26	2.70	3.90	4.65	2.74	3.26

	**at 520 nm**			**at max**			**at 640 nm**		
	$n_{DNA}^2 \times E_{520}$	$n_{water}^2 \times E_{520}$	n_{eff}	$n_{DNA}^2 \times E_{max}$	$n_{water}^2 \times E_{max}$	n_{eff}	$n_{DNA}^2 \times E_{640}$	$n_{water}^2 \times E_{640}$	n_{eff}
1.7 nm	7.85	7.29	1.445	11.90	11.04	1.444	8.01	7.48	1.449
3.3 nm	6.71	6.30	1.453	10.60	9.91	1.450	7.155	6.77	1.459
5 nm	5.78	5.45	1.456	9.50	9.07	1.466	6.48	6.30	1.478
6.7 nm	5.09	4.78	1.453	8.78	8.23	1.452	6.165	5.77	1.450

$$n_{eff} = n_{water}\sqrt{E_{water}/E_{DNA}}$$

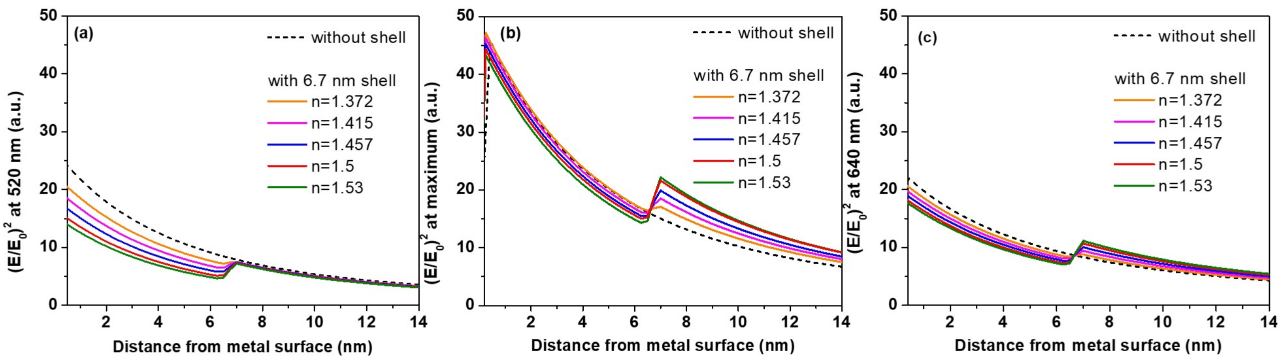

Figure S15. Decay of $(E/E_0)^2$ near plain 50 nm diameter AuNS and coated with 6.7 nm shell of different refractive indices, at 520 nm (a), 640 nm (c) and at the position of maximum field enhancement (at 553 nm for plain AuNS and at 557 nm for the AuNS + 6.7 nm shell (b).

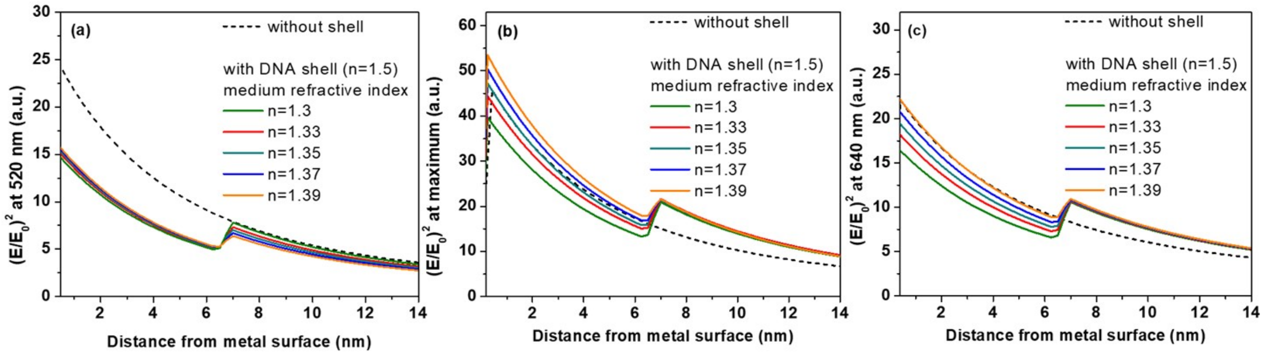

Figure S16. Decay of $(E/E_0)^2$ near plain 50 nm diameter AuNS and coated with 6.7 DNA shell (n=1.5) placed in a medium with refractive index in the 1.3-1.39 range, at 520 nm (a), 640 nm (c) and at the position of maximum field enhancement (at 553 nm for plain AuNS and at 557 nm for the AuNS + 6.7 nm DNA shell (b).
